# Influence of CaO on Physical and Environmental Properties of Granulated Copper Slag: Melting Behavior, Grindability and Leaching Behavior

**DOI:** 10.3390/ijerph192013543

**Published:** 2022-10-19

**Authors:** Lijun Sun, Yan Feng, Daolin Wang, Chongchong Qi, Xuemin Zeng

**Affiliations:** 1 State Key Laboratory of Safety and Health for Metal Mines, Maanshan 243000, China; 2 Sinosteel Group Maanshan Mining Research Institute Co., Ltd., Maanshan 243000, China; 3 School of Resource and Safety Engineering, Central South University, Changsha 410083, China

**Keywords:** granulated copper slag, thermochemically modification, leachability, melting behavior, solidification/stabilization

## Abstract

Due to its potential pozzolanic activity, granulated copper slag (GCS) has been proven to act as a supplementary cementitious material (SCM) after thermochemical modification with CaO. This modification method reduces cement consumption and CO_2_ emissions; however, the additional energy consumption and environmental properties are also not negligible. This paper aims to evaluate the economics and environmental properties of thermochemically modified GCS with CaO through the melting temperature, grindability, and heavy metal leaching characteristics. The X-ray fluorescence spectroscopy (XRF) results indicated that the composition of the modified GCS shifted to the field close to that of class C fly ash (FA-C) in the CaO-SiO_2_-Al_2_O_3_ ternary phase diagram, demonstrating higher pozzolanic activity. The test results on melting behavior and grindability revealed that adding CaO in amounts ranging from 5 wt% to 20 wt% decreased the melting temperature while increasing the BET surface area, thus significantly improving the thermochemical modification’s economics. The unconfined compressive strength (UCS) of the cement paste blended with 20 wt% CaO added to the modified GCS after curing reached 17.3, 33.6, and 42.9 MPa after curing for 7, 28, and 90 d, respectively. It even exceeded that of Portland cement paste at 28 d and 90 d curings. The leaching results of blended cement proved that the heavy metal elements showed different trends with increased CaO content in modified GCS, but none exceeded the limit values. This paper provides a valuable reference for evaluating thermochemically modified GCS’s economics and environmental properties for use as SCM.

## 1. Introduction

The large-scale development of the copper industry has contributed greatly to the world economy every year by providing copper raw materials [1]. With the economy’s rapid development, people’s desire for copper resources has become unprecedented [2]. However, the massive buildup of copper slag caused by the low grade of copper ore has presented significant environmental and solid waste management difficulties [3,4]. The rational resource utilization of copper slag, thus achieving environmental protection and clean production of copper is imminent [5].

In recent years, the manufacture of supplementary cementitious materials (SCM) using copper slag has attracted much attention from researchers [6]. In practice, this has proven to be the cheapest, simplest, and most cost-effective [7]. Copper slag as a partial substitute for cement is not only beneficial to reducing cement production and reducing greenhouse gas emissions but also can reduce the occupation of land resources [8,9,10]. Adding copper slag to concrete can improve aggregate compressibility and corrosion resistance, thereby increasing concrete’s durability. These improvements provide sustainable development to some extent and have multiple advantages [11]. However, the most significant difficulty is that granulated copper slag (GCS), the main form of copper slag, has relatively low pozzolanic activity, resulting in low utilization in cement and concrete [12].

To enhance the secondary utilization of GCS, researchers have made positive progress in enhancing the pozzolanic activity of GCS using mechanical, chemical, thermodynamic [13], and thermochemical [14] modification methods. Feng et al. [15] have indicated that adding CaO to the structure of GCS by thermochemical excitation can significantly advance the glass structure and pozzolanic activity of GCS. However, this research mainly focused on the structure, and little attention was paid to the economic costs and environmental effects arising from the modification.

Besides, under the current strict environmental protection policy, the leaching of heavy metals in composite cement containing GCS has attracted much attention for the use of GCS as SCM [16,17,18,19,20,21]. In addition to the principal oxides, there are also a small number of heavy metals in GCS, such as copper (Cu), zinc (Zn), and nickel (Ni) [19]. Although some scientists have shown that the contaminants extracted from composite cement containing copper slag and copper slag do not exceed the limit, these investigations are limited only to copper slag with low CaO (4%) content and composite binder with low cement substitution rate (10%) [22,23]. On the one hand, the structural properties of GCS changed due to the addition of the network modifier CaO [24]. This demonstrates that the leaching behavior of heavy metals in modified GCS may be affected. On the other hand, the thermal chemical modification of GCS also includes the melting and grinding stage, which inevitably requires energy consumption, affecting the application and promotion of this action method [25]. There are few studies on the effect of chemical modification on the leaching behavior of GCS and high cement replacement rate composite binders with different CaO content. In particular, it’s crucial to expand research into the costs and environmental impacts of thermochemically activating GCS to encourage its use in SCM [26]. Therefore, the main research of this work was to evaluate the physicochemical properties, grindability, mechanical behavior, and environmental properties of thermochemically modified GCS by CaO addition. 

## 2. Materials and Methods

### 2.1. Materials and Sample Preparation

GCS was provided by a copper smelter. Type I Portland cement (PC) used in this research was obtained from Heidelberg Cement AG. Reagent grade CaO (Nr. Eg: 215-138-9) from Alfa Aesar (Haverhill, MA, USA) was utilized as a chemical additive. Five different samples of GCS were prepared; one reference sample devoid of additives, and mixtures with 5, 10, 15, and 20 wt% CaO addition, respectively. Each of the GCS samples was melted in an alumina crucible using an induction furnace. The temperature was raised for the first 3 to 4 h at a rate of 100 °C/h and then continued to rise at a rate of 150 to 250 °C/h until the sample melted. Liquid slags were poured into cold water and the modified GCS samples with various CaO content were obtained for further analysis. The detailed sample preparation procedure is referenced in [27]. The samples with 0, 5, 10, 15, and 20 wt% CaO addition are named as MC0, MC5, MC10, MC15, and MC20, respectively. The modified GCS and PC chemical compositions were measured using X-ray fluorescence spectroscopy (XRF), and the results are shown in Table 1.

### 2.2. Melting Behavior

The melting behavior of the modified GCS was examined using a high-temperature microscope (HESSE-EM201) and characterized by the initial deformation temperature, softening temperature, melting temperature, and flow temperature. In this instance, the heating rate was held constant at 10 °C/min, and the maximum temperature was set at 1600 °C.

### 2.3. Grindability

The modified GCS was ground for two hours in a Humboldt Palla 20U high-performance vibratory ball mill with a vibration amplitude of 10 mm and frequency of 1000 rmp using cylindrical ceramic grinding balls as the grinding media Subsequently, the particles with particle sizes between 0.075 and 0.60 mm were screened by a JIS standard sieve and mixed well by using a sample splitter. The grindability of modified GCS samples was characterized by particle size and BET surface area [28]. The particle size distribution of the samples was tested by a particle size analyzer (CILAS 1064), and the BET surface area was defined by a nitrogen adsorber (Micromeritics FlowSorb II 2300).

### 2.4. Unconfined Compressive Strength (UCS) Test

The UCS test was used to characterize the mechanical behavior. The five samples of modified GCS were used to replace 30 wt% PC to form blended cement and were also named MC0, MC5, MC10, MC15, and MC20. Then, 50 wt% paste was prepared from the five blended cement and PC. The paste was poured into a cubic mold (4 × 4 × 4 cm) and was cured in a curing box of constant temperature (25 °C, 90% humidity) in which it solidified. The UCS values were measured by a SANS CDT1305 uniaxial electronic pressure tester at the curing ages of 7 d, 28 d, and 90 d. 

### 2.5. Leaching Test for Blended Cement

The leachability of the cement-based matrix is a crucial index to evaluate its immobilizing effect on heavy metals. The bulk paste samples were sieved through a 4 mm sieve to eliminate large particles. EN 12457-2 was used to determine the leaching of heavy metals from solidified/stabilized products (hardened pastes). Subsequently poured into plastic bottles together with deionized water set at a liquid-to-solid ratio of 10 L/kg. The bottles were located in a rotating agitator at 10 rpm for 24 h. The suspension was filtrated with a 0.45 μm filtration paper using a vacuum filtration device after being left to stand for 15 min. The leachate was sent to a certified laboratory for ICP (inductively coupled plasma) analysis. 

## 3. Results and Discussion

### 3.1. Characterization of the Modified GCS

**Figure 1:**
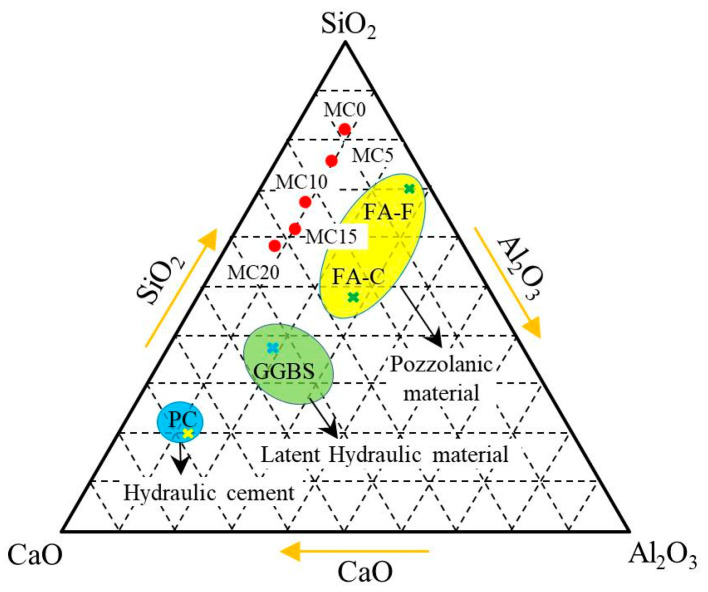
CaO–SiO_2_–Al_2_O_3_ ternary phase diagram (wt%). PC: Portland cement; GGBS: ground granulated blast furnace slag; FA-C: Class C fly ash; FA-F: Class F fly ash.

The chemical compositions of the modified GCS and PC samples are shown in Table 1. The main constituents present in modified GCS are FeO, SiO_2_, CaO, Fe_2_O_3_, and Al_2_O_3_, accounting for greater than 80 wt% of the total mass. It is common knowledge that cementitious materials can be classified as hydraulic cement, latent hydraulic material, and pozzolanic material, which are represented by PC, ground granulated blast furnace slag (GGBS), and fly ash (FA), respectively. SiO_2_, CaO, and Al_2_O_3_ are the main available components present in these cementitious materials. As such the CaO–SiO_2_–Al_2_O_3_ system is generally used to determine the suitability of pozzolanic materials when they are used as SCMs. The chemical compositions of the modified GCS samples in the CaO-SiO_2_-Al_2_O_3_ system are demonstrated in Figure 1. According to Figure 1, the original GCS has a substantially greater SiO_2_ content than CaO and Al_2_O_3_ and is located near Class F fly ash (FA-F) in the upper part of the ternary diagram. The composition of modified GCS shifts to the field around Class C fly ash as the CaO level is increased (FA-C). Antiohos et al. [29] evaluated the performance of blended cement with two different types of fly ash. As a result of the study, was indicated that FA-C exhibits higher pozzolanic activity than FA-F, owing to the higher CaO content. In light of the research, it appears that the pozzolanic activity of modified GCS is potentially improved by chemical modification with CaO. The modified GCS samples are centrally distributed on the ternary diagram of the CaO-SiO_2_-Al_2_O_3_ system, demonstrating well compatibility of CaO in the glass structure of the modified GCS [30]. 

Other metal oxide compounds and heavy metals (Cu, Zn, Cr, Sb, and Pb) in lower quantities are also present in the modified GCS. Although copper slag has been approved to be an inert waste by the European Union, the chemical modification with CaO may increase the leaching threat of heavy metals [19]. Therefore, the impact of CaO addition on the leaching of heavy metals from modified GCS should be taken into consideration when it is used as an SCM. 

### 3.2. The Influence of CaO Modification on Melting Behavior

**Figure 2:**
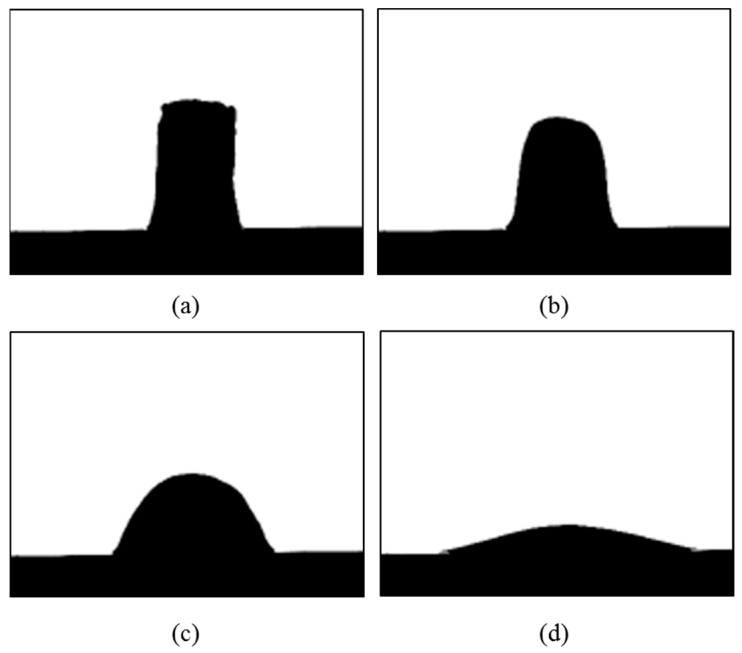
A typical series of images of the evolution of a slag cylinder with increasing temperature in a heating microscope, corresponding to (**a**) deformation temperature, (**b**) spherical temperature, (**c**) semi-spherical temperature, and (**d**) flow temperature.

The evolution of a slag sample with increasing temperature in a heating microscope is illustrated in Figure 2 using a typical series of video images that correspond to deformation temperature, spherical (softening) temperature, semi-spherical (melting) temperature, and flow temperature [31,32]. At the deformation temperature melting of the sample begins and shows early signals of softening [33]. For mixed samples, slag melts along with lime dissolution to form the initial liquid phase at this stage [34,35]. This liquid exists to fill the pores between particles resulting in the initial shrinkage [36]. With increasing temperature, the melting and dissolution proceed with the progressive depletion of solid particles. The slag cylinder exhibits an area change in expansion till slumping as the quantity of liquid steadily increases. Due to the varying degrees of lime incorporation into the structure, the composition of the liquid phase changes continuously during this fusing process until the sample is totally changed into a homogeneous liquid.

**Figure 3:**
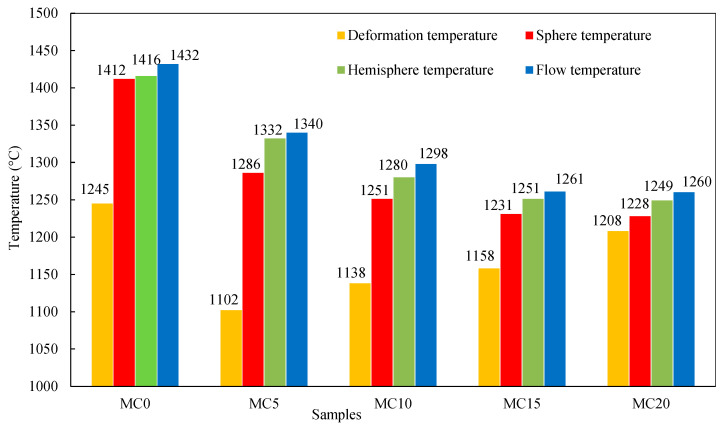
Characteristic temperatures of samples modified with CaO addition.

In Figure 3, the comparison of the characteristic melting temperatures for all samples is presented. It can be observed that the deformation temperature drastically decreased as the addition of CaO was increased to 5 wt%, on the other hand, with increases obviously with a further addition to 10 wt%. By reducing the liquid phase’s viscosity with the addition of CaO, the initial reduction may be attributed to the improvement in fluidity and enhanced diffusion of the liquid phase [37]. Nevertheless, the basicity of melt increases with the incorporation of lime, leading to a low rate of lime dissolution into FexO–CaO–SiO_2_ melt [38]. According to this phenomenon, undissolved lime particles increase the viscosity of molten slag [39], inhibiting its diffusion and increasing the deformation temperature. Due to the slow rates of liquid phase diffusion and the development of melt formation at relatively low temperatures, a significant gap can be identified between deformation and softening temperature for the MC0 sample. The viscosity and diffusion rate of the liquid phase considerably increased with the increase in temperature and lime’s dissolution into the molten slag, which resulted in a decreased difference between deformation and softening temperature.

Besides, lime addition is favorable to decreasing GCS’s softening, melting, and flow temperature. Especially the initial addition of 5 wt% CaO lowered these temperatures drastically. This trend became weaker as the addition of CaO was increased to 15 wt%. Each sample was completely transformed into a homogeneous liquid at flow temperature, and calcium acting as network modifiers was completely incorporated into the silicate melt structure. Compared to the reference sample, the flow temperature decrease could reach as high as 172 °C (nearly 12%) with a 20% CaO addition (MC20 sample). This can be attributed to the decrease in the degree of polymerization and total bond strength of the melt structure. For the additive process used in industrial manufacturing, the outcomes of melting behavior can be a valuable reference.

### 3.3. The Influence of CaO Modification on Grindability

**Figure 4:**
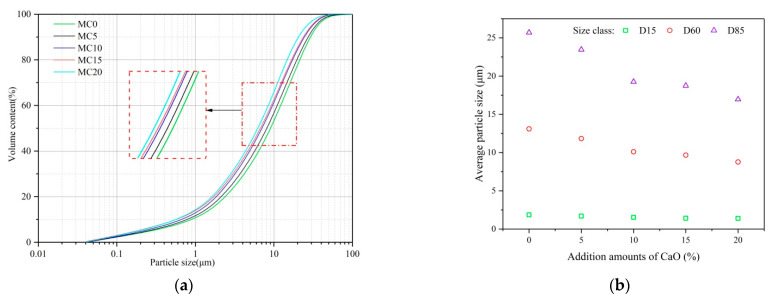
(**a**) Particle size cumulative distribution as a function of the addition amount of CaO (**b**) Characteristic particle sizes of tested samples.

For all modifying conditions, the particle size distribution analysis of GCS is presented in Figure 4. The findings showed that all samples exhibited a similar wide distribution of particle sizes, and as shown in Figure 4a, about 85% of the particles are less than 19 μm. The cumulative distribution curves were shifted to smaller diameters by increasing the amount of CaO addition into GCS. To quantitatively characterize the particle size distribution of modified GCS after grinding, D15, D60, and D85 were used with rates up to 15 wt%, 60 wt%, and 85 wt%, respectively.

Although the addition of CaO provided a slight decrease in D60 from 13.10 μm to 11.83 μm, a sharp reduction was seen in the subsequent 20 wt% (MC20) addition. Figure 4b shows the different samples’ characteristic diameters of D15, D60, and D85. The results showed that adding CaO to GCS could reduce the average particle size of the samples for different size classes, especially when the addition amount was increased up to 20 wt%. The increase in particle size was less pronounced for D15 than for the other characteristic diameters. Due to a change in the structural composition of GCS particles, the increasing effect of CaO addition on particle size reduction was primarily centered in the size classes from D60 to D85. No clear evidence demonstrating particle agglomeration was observed for different size classes in agreement with the investigation on the grinding for FA [40]. This is possibly associated with the vibration effect of the vibratory mill [41]. 

**Figure 5:**
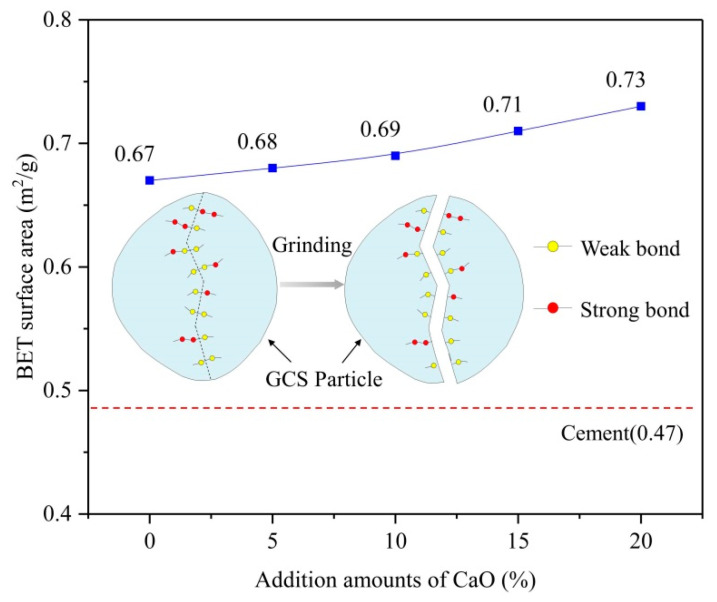
BET surface area of GCS as a function of the addition amount of CaO and selective breaking of weak bonds in GCS particles during grinding.

Figure 5 shows the variation of the specific surface area of ground slag as a function of the CaO addition amounts. BET surface area of all samples was found to be significantly higher than Portland cement, benefiting the packing effect on the performance of blended cement [42]. With increasing the content of CaO, the BET surface area of GCS increased gradually from 0.67 m^2^/g for the initial slag (MC0) to 0.73 m^2^/g for the modified slag of MC20 under the same grinding condition. The growth rate in the BET surface area of GCS was consistent with the variation of particle size distribution due to the increase in the number of fine particles. As a consequence of outcomes, CaO addition was noted favorable to improving the grindability of GCS. It has been demonstrated that the glass structure has a significant impact on the grindability of glassy materials [43].

According to the modified random mesh (MRN) model for glass structure proposed by Greaves [44], mesh modifiers and non-bridging oxygens (NBOs) exhibit a nonrandom and non-homogeneous distribution throughout the glass leading to segregated rich regions of modifiers, and large amounts of ionic bonds are present in these regions, which are much weaker than covalent bonds. As depicted in Figure 5, where GCS particle breakage occurs preferentially during grinding, weak interfaces may be formed due to the agglomeration of weak ionic bonds in modifier-rich regions [45]. As the number of network modifiers in glassy structures increased, the weak interfaces grew and developed in number and scope, resulting in the improvement of grindability. 

### 3.4. The Influence of CaO Modification on Mechanical Performance

**Figure 6:**
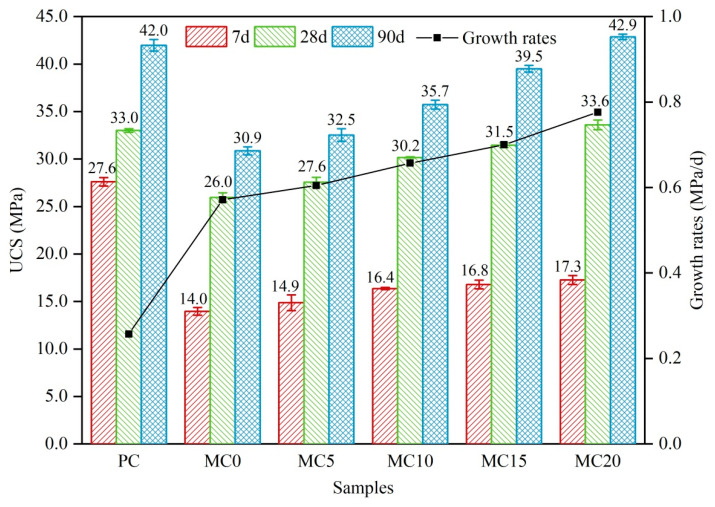
The UCS development of specimens prepared blended cement with different CaO additions at 7, 28, and 90 d.

Figure 6 demonstrates the UCS values at 7, 28, and 90 d and the strength growth rates from 7 to 28 d for PC and blended cement pastes. The results proved that the strength generation was significantly influenced by the age of curing and the type of GCS. It can be observed that the UCS values of all specimens showed an increasing trend with the extension of the curing period, although the growth rates varied. Many researchers have also confirmed that the cement-based hydration reaction continues during the 90-d curing period and produces more hydration products [12]. The CaO addition in the modified GCS was primarily responsible for the variation in strength amongst different composite cement samples under the same curing conditions and age. The strengths of MC20 samples at 7, 28, and 90 d were 17.3, 33.6, and 42.9 MPa, respectively, which were higher than the other blended cement samples at the same curing age, indicating that the CaO addition had a significant positive effect on the pozzolanic activity excitation of MGCS. Furthermore, it was found that, with the exception of the MC20 samples, all of the blended cement samples’ UCS values were lower than those of PC. Besides, this negative effect mainly resulted from the dilution effect of the cement, which diminished with the increase of CaO content in GCS.

The increasing acceleration of the GCS pozzolanic reaction, which results in the formation of a secondary C-S-H cementitious phase, explains why blended cement in its early stages displays lower strength values and stronger growth rates than PC. The weak early pozzolanic reaction plays a secondary role in the strength development of blended cement. Besides, it is insufficient to compensate for the negative results caused by the dilution effect. As the blended cement hydrates, the pozzolanic reaction gradually becomes the dominant factor in the effect of GCS on the strength of blended cement, resulting in an increased strength growth rate.

As the pozzolanic reaction proceeded, GCS continued to consume CH through the pozzolanic reaction, causing the pH of the pore solution to decrease. This slowed the reaction rate of GCS and reduced its impact on the development of later strength. The UCS value and development rate of blended cement from 28 to 90 d were considerably increased with the addition of CaO to GCS, revealing that the modification of GCS could enhance its pozzolanic activity and accelerate the development of compressive strength. Therefore, the GCS sample with the highest activity corresponding to the highest CaO addition (20 wt%) also had higher (MC20) strength values than other blended cement samples, and this advantage appeared especially at the later stage (28–90 d). The UCS value of MC20 reached a maximum (42.9 MPa) after 90 d of curing, which was higher than the PC sample (42.0 MPa).

### 3.5. Cement-Based Solidification/Stabilization 

Table 2 shows the leaching test results for blended cement pastes at 28 d. copper slag has been determined to be an inert waste by the European Union. The leaching of heavy metals is compared to the leaching limit values for waste acceptance at EU landfills, listed in Annex II to the Landfill Directive. None of the metal leachings from the cement-slag pastes exceeded the limiting values, of which potential threat comes from Cu and Ba in relatively high concentrations close to the regulatory limits. As a consequence, it can be stated that the majority of heavy metals in GCS can be securely stabilized in a cement-slag system. Further discussion will be focused on Ba, Cd, Cr, and Cu since the concentration of other contaminants is below the detection limits.

The leaching of the primary contaminants exhibited inconsistent trends for the different samples depending on their distribution areas in the cement-slag system. No obvious trend was observed for Cd due to its low concentration. An increment trend was observed for the leachable concentrations of Ba and Cr with increasing CaO content of GCS, which could be attributed to the higher dissolution rate resulting from GCS’s chemical modification. Additionally, this was consistent with the theory that Cr is present in the C-S-H matrix through adsorption and precipitation, which results in high leachable concentrations [46]. However, the opposite behavior was observed for Cu, where its concentration showed a slight decrease with the incorporation of CaO into GCS. This indicates the cement-based solidification’s influence on heavy metals’ leaching behavior.

Therefore, the cement system has a beneficial fixation/stabilization effect on GCS, and its use as supplementary cementitious material in cement products has no noteworthy adverse effects on the environment. Besides, some heavy metal elements showed different trends with the increased CaO content in GCS due to different corresponding fixation/stabilization mechanisms. 

## 4. Conclusions

The current study evaluated the effect of chemical modification with CaO addition on the pozzolanic activity of GCS in a composite binder with 30% cement replacement and its mechanical properties and leachability. The following conclusions can be drawn from this research:

1. The chemical modification of GCS by CaO addition can enhance its pozzolanic activity by improving the glass structure and supplying more abundant hydrated reactants;
2. The CaO addition to GCS significantly reduced the characteristic temperatures of the samples during melting, including deformation temperature, spherical (softening) temperature, semi-spherical (melting) temperature, and flow temperature. However, the addition of CaO up to 10 wt% (MC10) has been noted as a limit value, and the inclusion of an amount of CaO beyond this value did not result in the expected gain in GCS melting point reduction;
3. CaO addition facilitated the formation of weak interfaces in the glass structure, which improved the grindability of modified GCS. Among them, the MC20 sample presented the most superior grindability with a BET-specific surface area of 0.73 m^2^/g;
4. The compressive strength of the cement-slag pastes was similar at low values after 3 and 7 d under all modifying conditions. The compressive strength of the paste samples increased as the CaO content of GCS increased, especially at later ages (28 d and 90 d). As the amount of CaO addition was increased to 20%, the cement-slag paste strength was comparable to the reference cement paste at 28 d, and outperformed at 90 d;
5. For the heavy metals, the limiting values were not surpassed by any of the metal leachings from the cement-slag pastes. The leaching of the main contaminants (Ba, Cd, Cr, and Cu) exhibited inconsistent trends with increasing CaO content in GCS due to their distribution areas in the cement-slag system.

## Figures and Tables

**Table 1:** Chemical composition of the modified GCS and PC (wt% by weight).

Samples	FeO	SiO_2_	CaO	Fe_2_O_3_	Al_2_O_3_	MgO	SO_3_	Zn	Cu	Cr	Sb	Pb
MC0	35.89	33.40	4.00	7.14	3.50	1.39	–	1.08	0.74	0.29	0.04	0.02
MC5	34.21	32.80	9.10	6.84	4.80	0.95		1.06	0.60	0.28	0.04	0.01
MC10	32.18	33.30	12.30	6.41	4.60	1.12	–	1.02	0.69	0.27	0.04	0.02
MC15	31.95	33.10	16.50	6.25	4.50	1.45		0.99	0.61	0.28	0.04	0.01
MC20	31.31	32.30	19.50	6.23	3.90	0.85	–	0.94	0.62	0.26	0.04	0.02
PC	–	18.10	62.10	2.80	4.90	1.20	3.70	–	–	–	–	–

**Table 2:** Leachability of different heavy metals from cement-slag pastes at 28 d.

Element	MC0 (mg/L)	MC5 (mg/L)	MC10 (mg/L)	MC15 (mg/L)	MC20 (mg/L)	Limiting Values (mg/L)
As	—	—	—	—	—	0.500
Ba	15.632	16.158	16.997	16.549	16.279	20.000
Cd	0.010	0.008	0.006	0.010	0.012	0.040
Cr	0.059	0.061	0.065	0.069	0.071	0.500
Cu	0.186	0.168	0.155	0.155	0.153	2.000
Ni	—		—		—	0.400
Pb	—		—		—	0.500
Sb	—		—		—	0.060
Zn	—		—		—	4.000

The values in the table are accurate to 0.01, with three decimal places.

## Data Availability

Not applicable.
